# P-116. Impact of a gastrointestinal molecular panel in the tropics: going beyond *E. histolytica*

**DOI:** 10.1093/ofid/ofae631.323

**Published:** 2025-01-29

**Authors:** Alana M Agramonte-Medrano, Anel E Guzman-Marte, Rita A Rojas-Fermin, Ann S Sanchez-Marmolejos, Maria Fernanda Cedeño-Bruzual, Lia Michelle Chaddy Báez, Dolores Magdalena Mejia De La Cruz

**Affiliations:** Hospital General de la Plaza de la Salud, Distrito Nacional, Distrito Nacional, Dominican Republic; Hospital General de la Plaza de la Salud, Distrito Nacional, Distrito Nacional, Dominican Republic; Hospital General de la Plaza de la Salud, Distrito Nacional, Distrito Nacional, Dominican Republic; Hospital General Plaza de la Salud, Santo Domingo, Distrito Nacional, Dominican Republic; Hospital General de la Plaza de la Salud, Distrito Nacional, Distrito Nacional, Dominican Republic; Hospital General de la Plaza de la Salud, Distrito Nacional, Distrito Nacional, Dominican Republic; Hospital General de la Plaza de la Salud, Distrito Nacional, Distrito Nacional, Dominican Republic

## Abstract

**Background:**

Acute diarrhea (AD) is a major cause of illness and hospital admissions, particularly in humid tropical climates. These infections can escalate rapidly, making an early and accurate diagnosis crucial for effective treatment. However, due to the variety of pathogens and the limitations of conventional diagnostic tests, clinicians often resort to empiric treatments based on epidemiological data. This study explores how effective a gastrointestinal molecular panel (GI panel) can be in identifying specific pathogens in a tertiary health center.

**Methods:**

This observational, descriptive, cross-sectional study analyzed the clinical records of patients over 18 years old with symptoms of AD. These patients underwent treatment and took the FilmArray GI Panel between March 2021 and December 2022. The study also compared the results obtained by ova and parasite tests and/or stool culture, when available.

Viral, Bacterial & Parasitic infections

**Figure:**
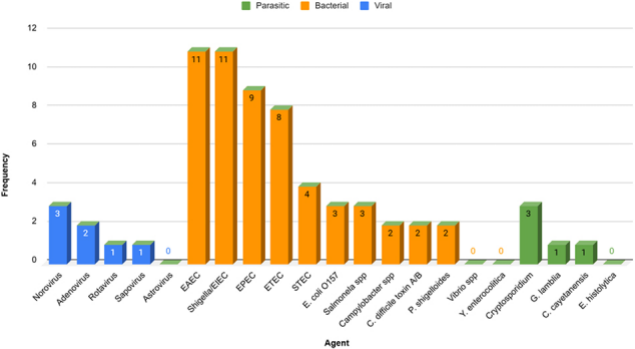

Source: Statistical registry of the authors

**Results:**

Out of 59 patients (median age: 48 years; 57.63% female), the GI panel identified at least one pathogen in 66.1% (39/59) of samples, with 32.2% (19/59) showing two or more pathogens. Bacteria were the most common cause of infection (62.7%), with enteroaggregative *E. coli* (18.6%), *Shigella*/enteroinvasive *E. coli* (18.6%), and enteropathogenic *E. coli* (15.3%) being the most prevalent. Viruses accounted for 11.9% of cases, with Norovirus GI/GII (5.1%) and Adenovirus F 40/41 (3.4%) being the most common. Protozoa made up 6.8% of cases, primarily *Cryptosporidium* (5.1%) and *Cyclospora cayetanensis* (1.7%).

Out of 21 patients with a bacterial target in the GI panel, only one received a positive stool culture (Salmonella sp.). The 4 protozoa-positive samples were not detected through an ova and parasite test, and no stool sample was positive for *Entamoeba histolytica* (32 ova and parasite tests).

**Conclusion:**

It is necessary to rethink the etiology of AD in low- and middle-income countries like the Dominican Republic, which has long been based on low-sensitivity routine tests. Despite being in the tropics, protozoa were not the most frequent pathogens, and *E. histolytica* was largely absent, regardless of the diagnostic method used. Diagnostic routine tests should be revised to include targets that may be missed due to method limitations.

**Disclosures:**

**Rita A. Rojas-Fermin, MD,FIDSA**, Gilead: Advisor/Consultant|Pfizer: Advisor/Consultant|Pfizer: Honoraria

